# Role swapping between nurses and physicians in simulated endoscopy training enhances collaboration and teamwork: a prospective observational pilot study

**DOI:** 10.1097/MEG.0000000000003051

**Published:** 2025-08-05

**Authors:** Federica Borrelli de Andreis, Salvatore F. Vadalà di Prampero, Ilaria Simonelli, Francesca Motta, Silvana Fusha, Francesco Esposito, Alessia Mancini, Anna Di Gemma, Guido Costamagna, Milutin Bulajic

**Affiliations:** a Centre of Excellence of Gastrointestinal and Metabolic Diseases, Digestive Endoscopy Unit – Isola Tiberina – Gemelli Isola Hospital; b Catholic University of Sacred Heart; c Biostatistical Service, Clinical Trial Center; d Clinical Psychology Unit, Isola Tiberina – Gemelli Isola Hospital; e Department of Dynamic and Clinical Psychology, Health Psychology School, Sapienza University of Rome, Rome, Italy

**Keywords:** interdisciplinary communication, patient care team, simulation training

## Abstract

**Background and aims:**

Gastrointestinal endoscopy is a collaborative process requiring technical and nontechnical skills from both physicians and nurses. Role swapping during simulated procedures has the potential to enhance skill development and team dynamics. The study aimed to evaluate the impact of role swapping on technical and nontechnical skills, as well as on team collaboration and satisfaction, among endoscopists and nurses during simulated endoscopic procedures.

**Methods:**

A pilot study was conducted with 22 participants (10 endoscopists and 12 nurses). Participants completed pre- and postsimulation assessments using validated self-rating scales: Non-Technical Skills for Surgeons (NOTSS), Scrub Practitioners’ List of Intraoperative Non-Technical Skills (SPLINTS), and customised technical skills questionnaires. Role swapping training sessions included onsite classes, randomised role-swapping simulations, and a 2-week postsimulation assessment. Wilcoxon nonparametric tests assessed differences between pre- and postsimulation scores.

**Results:**

Role swapping significantly improved NOTSS and SPLINTS scores across key domains: communication, decision-making, situational awareness, and teamwork (*P* < 0.05). Endoscopists reported significant confidence gains in instrument preparation, medication dilution, and patient discharge (*P* < 0.05). Nurses demonstrated improvement in motor skills, mucosal inspection, and loop reduction handling during colonoscopy (*P* < 0.05). Both groups expressed high satisfaction with role swapping training.

**Conclusion:**

Role swapping in simulated settings significantly enhances technical and nontechnical skills, fostering teamwork and mutual respect between physicians and nurses. This innovative approach could improve clinical practice and patient safety in real-world settings.

## Introduction

Gastrointestinal endoscopy is a cornerstone in the diagnosis and treatment of gastrointestinal diseases. With its growing demand worldwide, it remains a complex practice that requires not only advanced technical expertise but also well-developed nontechnical skills, such as communication, teamwork, decision-making, and situational awareness to ensure procedural success and patient safety [1,2].

Therefore, high-quality training in gastrointestinal endoscopy is indispensable, even for basic procedures such as oesophagogastroduodenoscopy and colonoscopy. Trainees need to develop cognitive skills, master different tools, and foster effective communication within a team. Moreover, they must integrate all these competencies beyond the technical procedure into a patient-centred management plan. Despite its importance, basic gastrointestinal endoscopy training has not yet been standardised across many healthcare systems.

Addressing this gap, the European Society of Gastrointestinal Endoscopy and the European Society of Gastroenterology and Endoscopy Nurses published their first position statement on basic gastrointestinal endoscopy in 2024 [2]. The document also acknowledges the importance of a multidisciplinary team working together to integrate a broad set of skills for optimal outcomes.

Though several studies have recognised the importance of collaboration in gastrointestinal endoscopy, there is no specific guidance on achieving this in practice. Traditional training methods remain largely individualistic and rarely provide opportunities for cross-disciplinary skill enhancement.

In recent years, role swapping has been explored as a potential solution in simulated settings [3]. Role swapping involves team members temporarily exchanging roles, allowing them to experience and understand the challenges and responsibilities of their colleagues. This approach has proven promising in fostering mutual understanding, promoting skill development, and enhancing teamwork [4,5]. While studies on role swapping have primarily focussed on surgical contexts, its application to gastrointestinal endoscopy training has not been explored yet.

This study aimed to evaluate whether role swapping during a simulated gastrointestinal endoscopy training could improve both technical and nontechnical skills among physicians and nurses. In addition, the study sought to assess changes in role awareness, mutual respect, and overall satisfaction with the training method.

## Materials and methods

### Study design

This is a pilot, prospective observational study using physical silicone simulators for colonoscopy training, where physicians and nurses underwent structured role-swapping sessions.

**Fig. 1.**
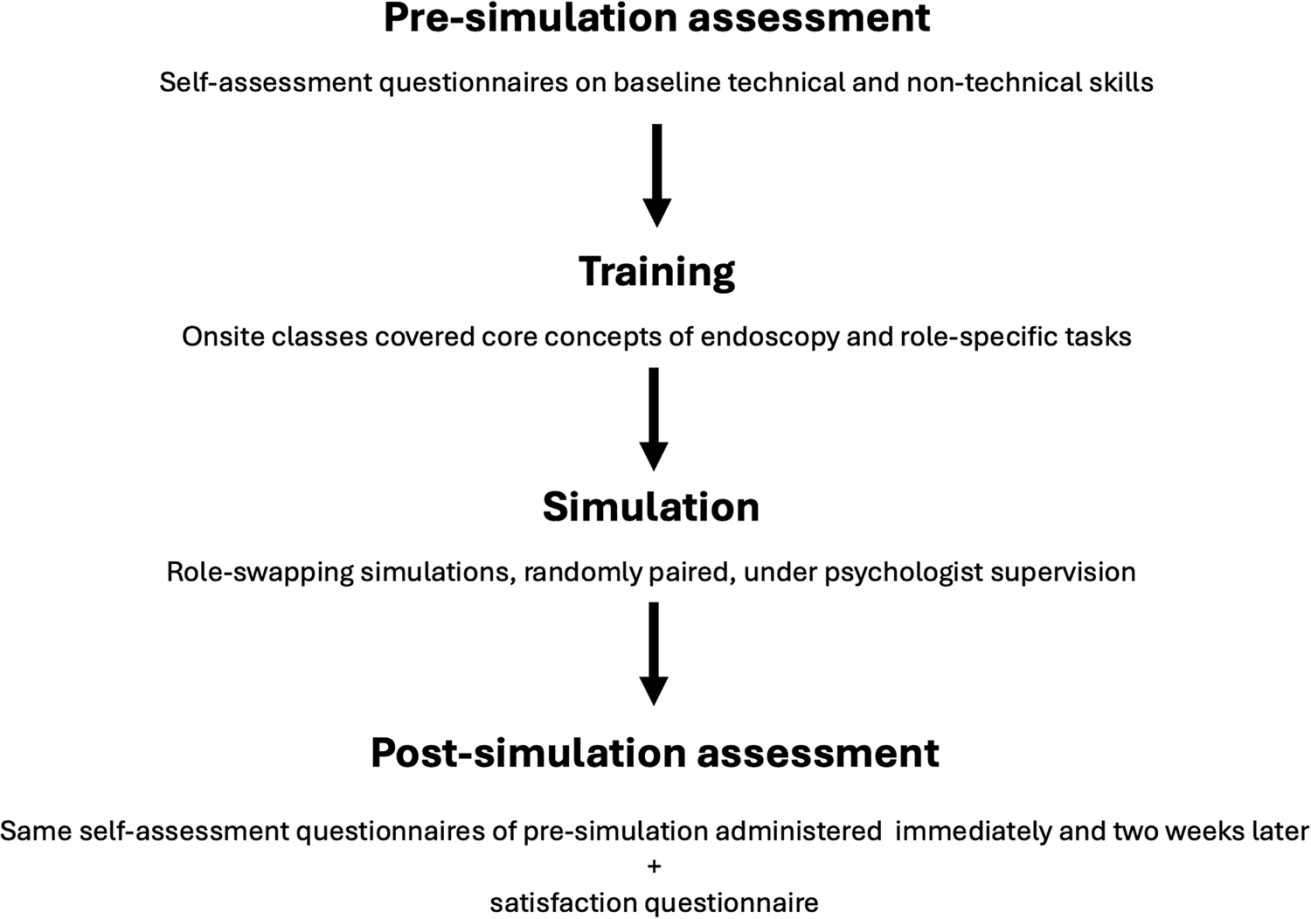
Study design.

The study design consisted of four phases (Fig. 1):

1. Presimulation assessment: Participants completed self-assessment questionnaires to measure baseline technical and nontechnical skills.
2. On-site theoretical classes: Participants then attended on-site classes covering core concepts of basic endoscopy and role-specific tasks:
    a.  Nurses instructed physicians on nursing tasks (e.g. patient positioning, monitoring vital signs, ancillary manoeuvres, etc.).
    b.  Physicians explained the technical tasks involved in endoscopy (e.g. scope navigation, forceps biopsy technique, polyp removal, etc.).
3. Simulation phase: Participants engaged in role-swapping exercises and were randomly paired to simulate real-world conditions. Physicians performed nursing tasks while nurses handled the endoscope.
4. Postsimulation assessment: Participants completed postsimulation satisfaction forms immediately after the simulation and follow-up evaluations 2 weeks later to assess skill retention and clinical application.

This study was conducted in accordance with the ethical principles of the Declaration of Helsinki. All participants provided informed consent. Participation was voluntary, and responses were anonymised to ensure confidentiality.

### Participants

We included 22 healthcare professionals (10 endoscopists and 12 nurses) actively involved in daily endoscopy practice. The inclusion criteria included licensed physicians and nurses with at least 3 months of experience in gastrointestinal endoscopy, who provided informed consent to participate in the study. Exclusion criteria were: lack of consent to participate and insufficient experience in either nursing or endoscopy to safely join the simulation. All participants were randomised using a computer-based system and paired for the simulation, with each endoscopist randomly assigned to a nurse.

### Simulation phase

The simulation-based gastrointestinal endoscopy was conducted using the physical silicone colonoscopy trainer (CM-15 Colonoscopy Simulator; Olympus Medical Systems Corporation, Tokyo, Japan). Under the supervision of an experienced endoscopist, a nurse performed a simulated colonoscopy, attempting basic procedures such as biopsies and simple polypectomies in a controlled, simulated environment. Simultaneously, an endoscopist, guided by a nurse, prepared the endoscopy room, monitored the patient, and managed the instruments during the simulated colonoscopy. The simulation session lasted approximately 30 min for each couple, ensuring participants experienced both roles within the endoscopic team in a realistic setting. A psychologist specialised in training and education passively observed the simulation to monitor participant behaviour and interaction.

### Tools and measures

The following questionnaires were used to assess participants’ skills and satisfaction (see Supporting Information Materials, Supplemental digital content 1, https://links.lww.com/EJGH/B205 for more details).

1. Technical Skills Questionnaires: Self-administered questionnaires assessing confidence and competence in role-specific tasks in gastrointestinal endoscopy (Supplementary Tables S1 and S2, Supplemental digital content 1, https://links.lww.com/EJGH/B205). Because of the lack of existing instruments specifically designed to assess technical skills in role-swapping endoscopy scenarios, we developed ad hoc questionnaires based on current guidelines on basic endoscopy and expert consensus within the research team (including senior endoscopists and nurse trainers) [2,6,7].
2. Non-Technical Skills for Surgeons (NOTSS) [8]: A validated tool evaluating nontechnical skills such as communication, decision-making, teamwork, and situational awareness for physicians (Supplementary Table S3, Supplemental digital content 1, https://links.lww.com/EJGH/B205).
3. Scrub Practitioners’ List of Intraoperative Non-Technical Skills (SPLINTS) [9]: A validated tool evaluating nontechnical skills such as communication, decision-making, teamwork, and situational awareness for nurses (Supplementary Table S4, Supplemental digital content 1, https://links.lww.com/EJGH/B205).
4. Satisfaction Questionnaire: Measuring participants’ appreciation, perceived value, and relevance of the simulation experience.

All the participants completed the technical skills questionnaires (one for nurses and one for endoscopists), NOTSS, and SPLINTS forms before the theoretical lessons and simulation to establish baseline levels. Immediately after the simulation, participants filled out the Satisfaction Questionnaire. Finally, 2 weeks after the simulation, participants repeated the technical skills questionnaire, NOTSS, and SPLINTS tools to evaluate skill retention and application in clinical practice. Responses were recorded in online questionnaires (Google Forms).

### Outcomes

The primary outcome was the improvement in nontechnical skills, including communication, teamwork, decision-making, and situational awareness. These skills were assessed through standardised observational tools (NOTSS and SPLINTS forms [8,9]) before and after the simulated endoscopy sessions.

The secondary outcomes were:

1. The improvement of technical skills for both endoscopists and nurses, including scope navigation for nurses and patient care protocols for physicians.
2. Overall satisfaction was assessed using a dedicated postsimulation satisfaction questionnaire, which measured perceived value, relevance, and enjoyment of the role-swapping experience.

### Statistical analysis

Descriptive statistics, such as median and interquartile range, were calculated for all ordinal variables, such as technical and nontechnical skills scores. Categorical variables like satisfaction levels were summarised using frequencies and percentages. Pre- and postsimulation scores for both technical and nontechnical skills were compared using a nonparametric Wilcoxon test for paired samples. A *P* value less than 0.05 was considered statistically significant. Box plots were used to display pre- and postsimulation data of each item of the technical skills questionnaires for endoscopists and nurses, respectively. Radar plots were applied to visualise the pre- and postsimulation ratings of each question of the NOTSS test and the SPLINTS, respectively. Data analysis was performed using the statistical software SPSS v9.

## Results

In October 2024, 22 participants, including 10 endoscopists (30% female, with a median experience in gastrointestinal endoscopy of over 5 years and a median working hours per week of <40 h) and 12 nurses (75% female, with a median experience of 5 years or less and a median working hours per week of <40 h), took part in this pilot study (Table 1).

**Table 1.** Study population

	Total participants (*N* = 22)
Classes of age	
<30 years, *n* (%)	6 (27%)
30–39 years, *n* (%)	5 (23%)
40–49 years, *n* (%)	4 (18%)
50–59 years, *n* (%)	5 (23%)
≥60 years, *n* (%)	2 (9%)
Male sex, *n* (%)	10 (45%)
Experience in endoscopy	
<6 months, *n* (%)	1 (5%)
6 months to 1 year, *n* (%)	6 (27%)
1–3 years, *n* (%)	4 (18%)
3–5 years, *n* (%)	4 (18%)
>5 years, *n* (%)	7 (32%)
Role	
Endoscopist, *n* (%)	10 (45%)
Nurse, *n* (%)	12 (55%)
Weekly working hours in the endoscopy room	
<20 h, *n* (%)	3 (14%)
≥20–29 h, *n* (%)	5 (23%)
≥30–<40 h, *n* (%)	14 (64%)

### Improvements in nontechnical skills

**Fig. 2.**
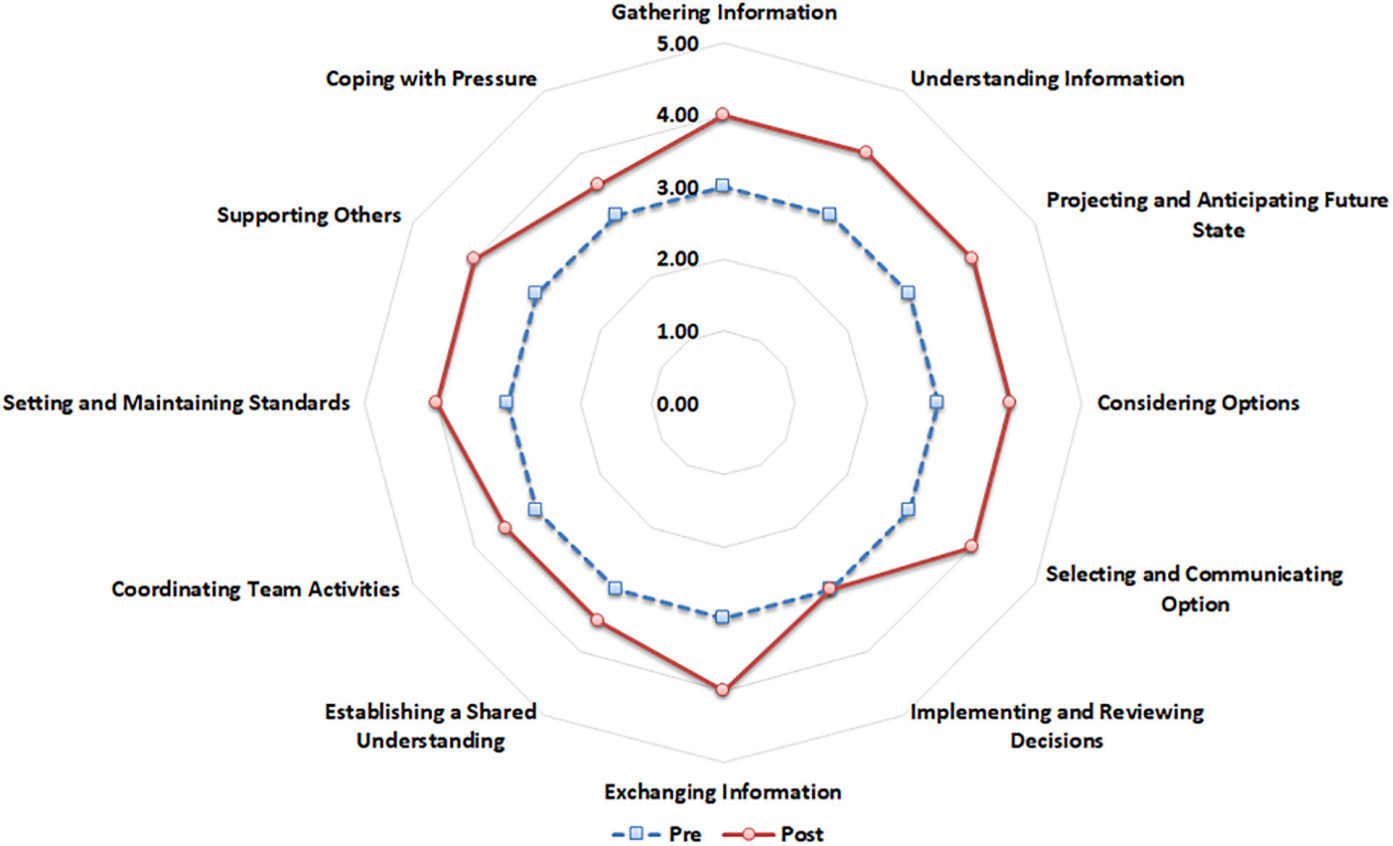
Radar plot of the Non-Technical Skills for Surgeons test in the pre- and postsimulation assessment for both endoscopists and nurses. The plot highlights changes in nontechnical skills across key domains.

**Fig. 3.**
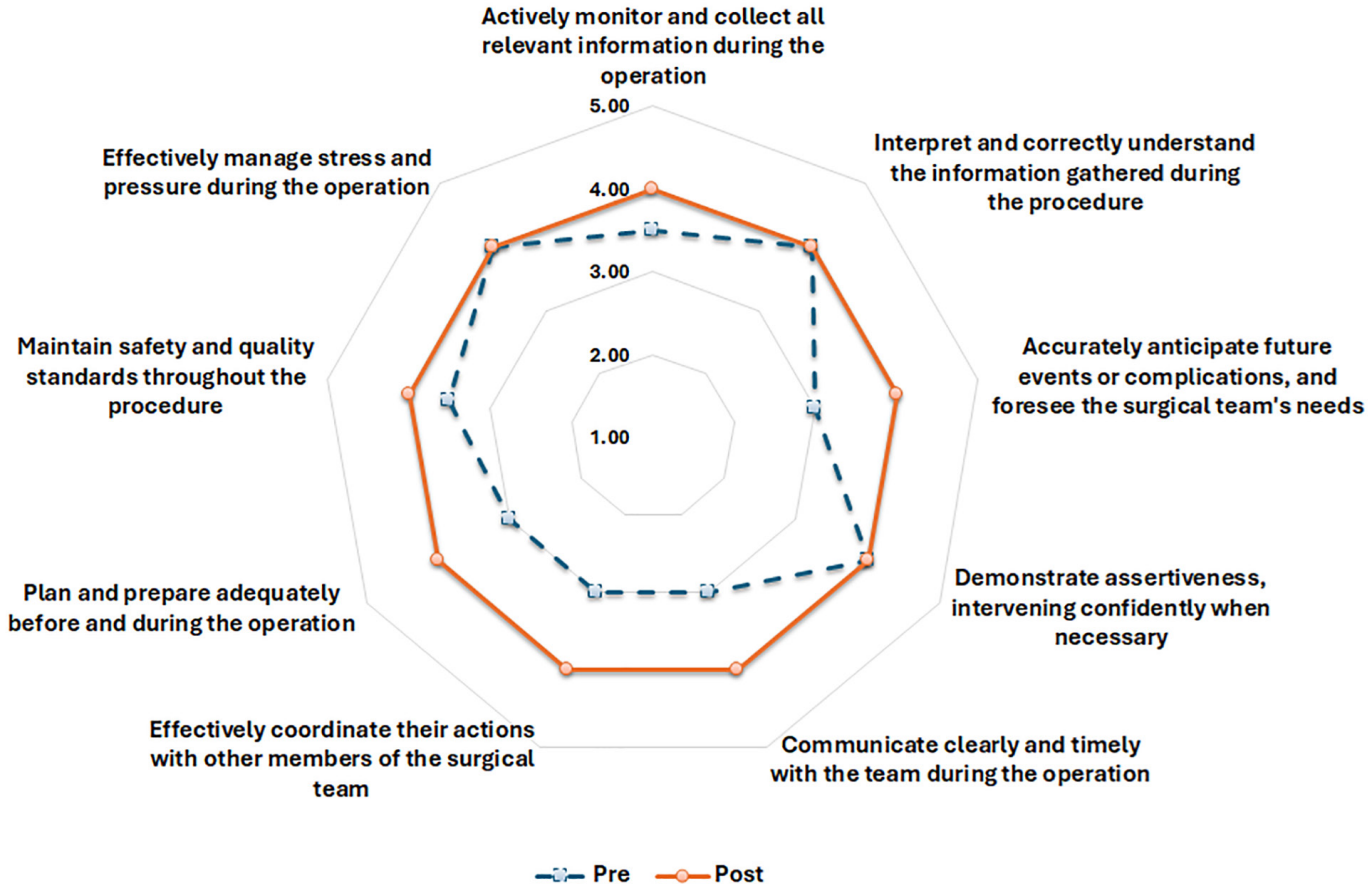
Radar plot of the Scrub Practitioners’ List of Intraoperative Nontechnical Skills questionnaire in the pre- and postsimulation assessment for both endoscopists and nurses. The plot highlights changes in nontechnical skills across key domains.

Statistically significant improvements were observed across all domains evaluated by the NOTSS (Fig. 2) and SPLINTS (Fig. 3) frameworks. Key areas of improvement included situation awareness, decision-making, communication, teamwork, and leadership. Specifically, notable enhancements were recorded in gathering and understanding information, as well as projecting future scenarios, with statistically significant results for information gathering (*P* = 0.02), understanding (*P* = 0.008), and future state projection (*P* = 0.002). In decision-making, there was a marked improvement in participants’ ability to consider options (*P* < 0.001), communicate decisions (*P* = 0.001), and adapt to changing conditions (*P* < 0.001). Communication and teamwork showed significant improvements, including improved information exchange (*P* = 0.007), shared understanding (*P* = 0.035), and team coordination (*P* = 0.001). In addition, leadership skills were strengthened, particularly in setting standards (*P* = 0.002) and supporting team members (*P* = 0.012).

### Technical skills development

**Fig. 4.**
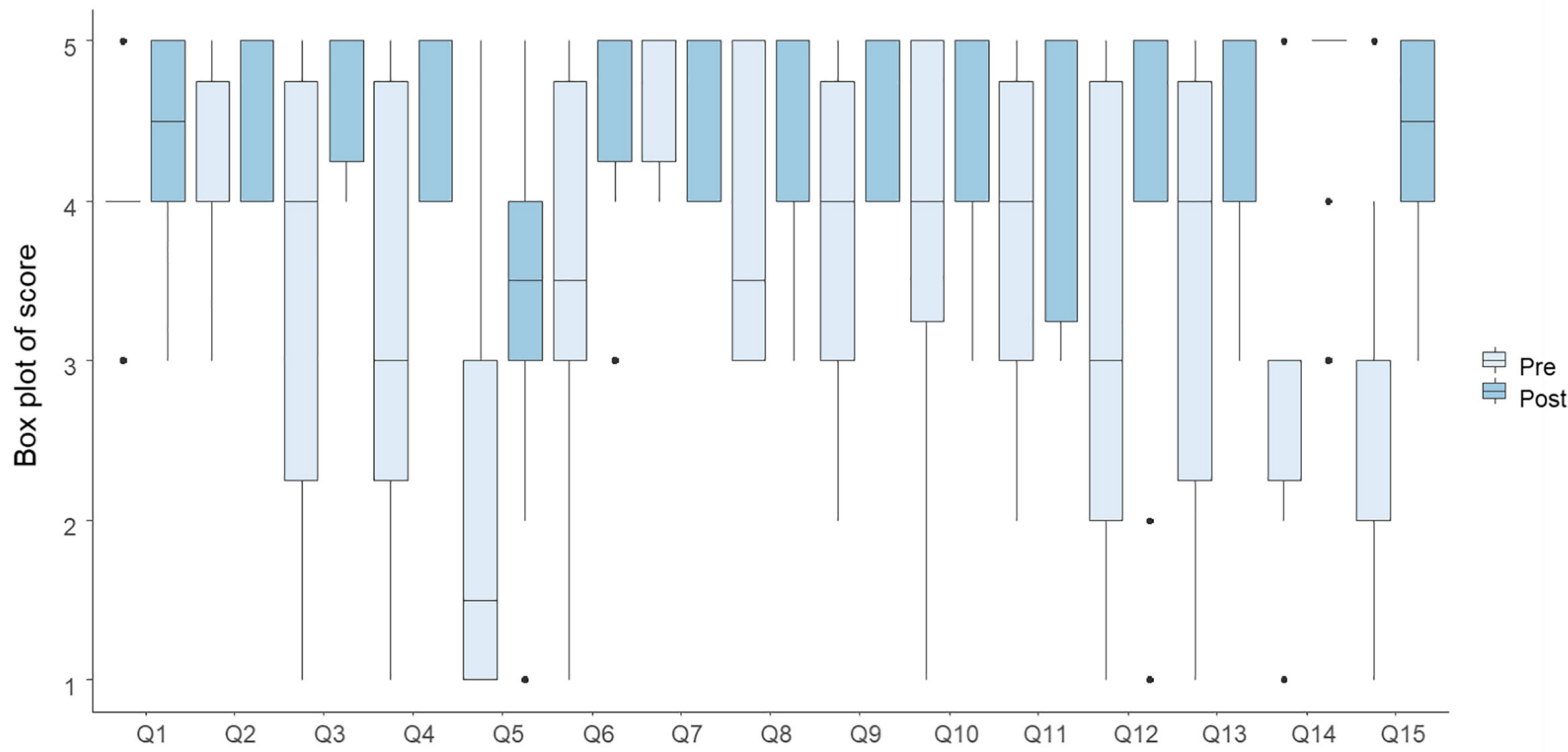
Box plot showing pre- and postsimulation confidence levels of endoscopists in performing nurse-related technical skills during colonoscopy. The plot illustrates the median, interquartile range, and score variability for each skill (Q1–Q15). For further details, please see the Supporting Information Materials, Supplemental digital content 1, https://links.lww.com/EJGH/B205.

Regarding technical skills development, the training simulation substantially improved endoscopists’ confidence across several critical areas. Notable advancements were observed in preparing endoscopic equipment, including the endoscopic tower, where confidence scores increased significantly (*P* = 0.016). Participants also showed a marked enhancement in their ability to prepare and dilute medications, significantly improving confidence levels (*P* = 0.016). Furthermore, managing patient discharge procedures saw a notable rise in confidence, with posttraining scores reflecting a median increase (*P* = 0.014). Additional areas of significant progress included arranging instruments during interventional colonoscopies (*P* = 0.026) and setting up accessories for monitoring patients’ vital signs before procedures (*P* = 0.041). Even tasks that initially exhibited lower presimulation confidence, such as disposing of waste after a colonoscopy, showed substantial improvements (*P* = 0.01) (Fig. 4).

**Fig. 5.**
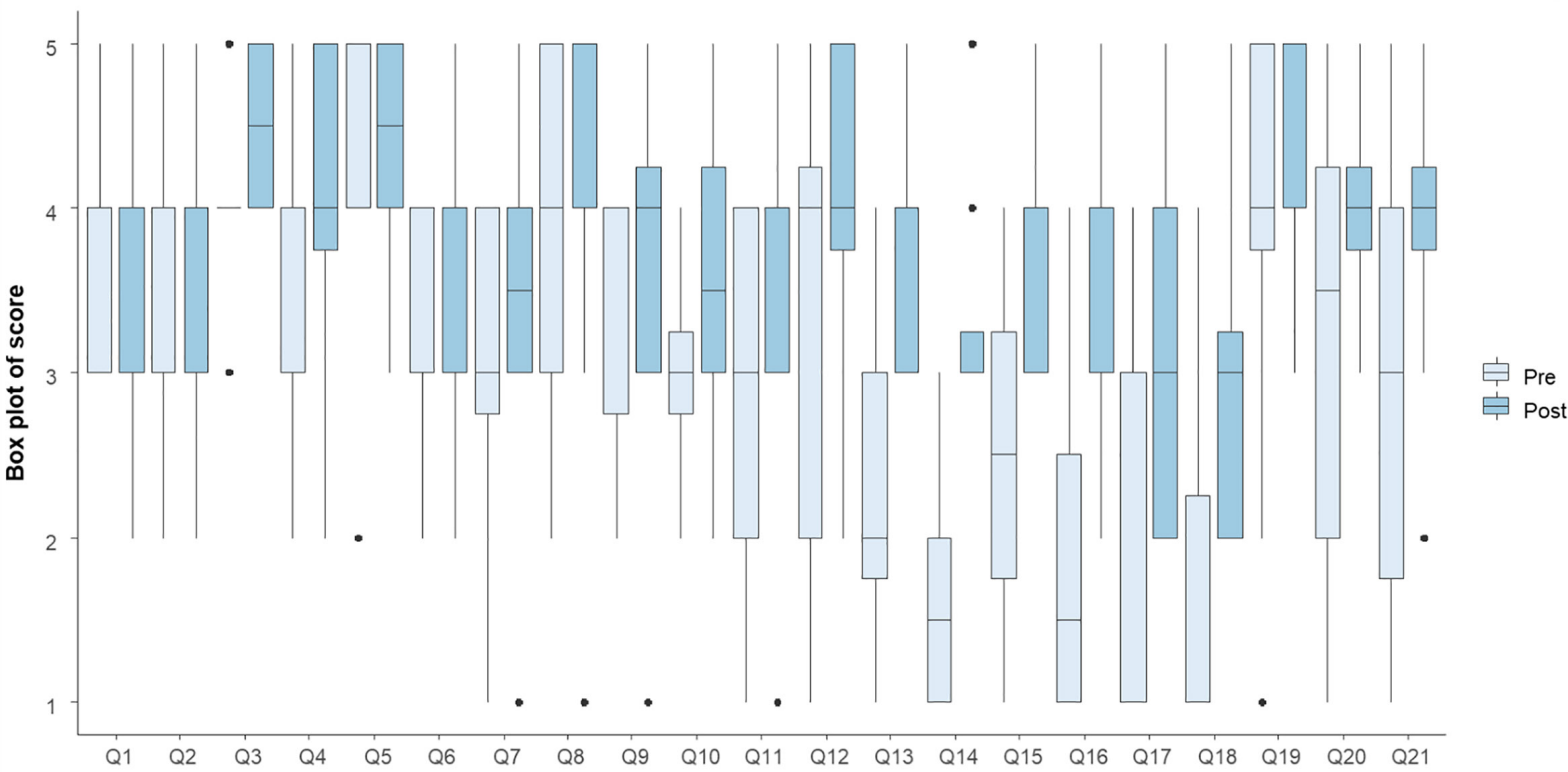
Box plot showing pre- and postsimulation confidence levels of nurses in performing endoscopist-related technical skills during colonoscopy. The plot illustrates the median, interquartile range, and score variability for each skill (Q1–Q21). For further details, please see Supporting Information Materials, Supplemental digital content 1, https://links.lww.com/EJGH/B205.

Nurses showed significant improvements in key technical skills after simulation training, including advancing the colonoscope (*P* = 0.004), reducing loops (*P* = 0.007), and navigating angled turns (*P* = 0.007). Their confidence also increased in performing ancillary manoeuvres (*P* = 0.038), mucosal inspection during withdrawal (*P* = 0.036), motor skills (*P* = 0.039), and colonoscope insertion (*P* = 0.026). In addition, they demonstrated a better understanding of colonoscopy quality indicators (*P* = 0.011) and an improved ability to identify pathological lesions (*P* = 0.046) (Fig. 5).

### Participant satisfaction

Both the endoscopists and nurses reported high levels of satisfaction with the role swapping training experience. The training was perceived to have boosted their confidence in collaborative practices and fostered a greater appreciation for each other’s roles within the team (Supplementary Fig. S1, Supplemental digital content 1, https://links.lww.com/EJGH/B205).

## Discussion

This pilot study aimed to explore the potential benefits of role swapping in endoscopic training, specifically its impact on the development of technical and nontechnical skills among endoscopists and nurses. The results demonstrated significant improvements in various key areas, suggesting that role swapping could enhance collaborative practices and foster mutual respect among team members.

A notable finding of this study was the improvement in nontechnical skills, as assessed by the NOTSS and SPLINTS frameworks. Specifically, the areas of situation awareness, decision-making, communication, and teamwork marked progress. Improvements in situation awareness, including the ability to gather and understand information, as well as to anticipate procedural developments, suggest that role swapping can provide participants with a deeper understanding of the complexities involved in clinical decision-making. These findings are consistent with existing research that underscores the importance of situational awareness in high-stakes environments, such as endoscopy, where patient safety can be directly influenced by the team’s ability to react to dynamic and unpredictable scenarios [10].

Role swapping may also promote better communication and teamwork between professional roles. In our study, participants demonstrated better information exchange, a stronger shared understanding, and enhanced team coordination. Effective communication has been widely recognised as one of the pillars of patient safety and procedural success [11], and the improvements observed in this study suggest that role swapping provides an opportunity to break down professional silos, encouraging open dialogue and collaboration across disciplines. This is particularly important in settings such as gastrointestinal endoscopy, where physicians and nurses must work closely together to ensure patient safety and procedural efficacy.

Another key outcome was the observed improvement in leadership skills, particularly in setting standards and supporting team members. Leadership in endoscopy is not solely the responsibility of senior physicians but requires contributions from all team members. The development of leadership skills across all participants suggests that role swapping may help cultivate a more collaborative and distributed leadership model within healthcare teams, which could have long-term benefits for procedural outcomes and team dynamics [12].

In terms of technical competence, simulation-based education using a physical silicone colonoscopy trainer has proven to be a valuable tool for endoscopy training, allowing realistic practice without the risks of in-vivo settings [13]. Both endoscopists and nurses demonstrated technical improvements in their usual scope of practice. For endoscopists, the role swapping sessions increased confidence in preparing endoscopic equipment, managing medications, and discharging patients. These skills are critical for ensuring smooth procedural operations and minimising patient risks, and their enhancement suggests that role swapping could be an effective method for refining these technical tasks. Similarly, nurses demonstrated significant progress in colonoscopy-related skills, including advancing the colonoscope, reducing loops during the procedure, and navigating angled turns. Providing nurses with opportunities to practice tasks beyond their primary responsibilities enhances their understanding of procedural workflows and fosters greater collaboration. By gaining a deeper understanding of the challenges faced by their colleagues, nurses may be better positioned to anticipate needs and provide more effective support during procedures [14].

Several studies in surgical settings have already demonstrated the value of role swapping in improving team dynamics and collaboration. For example, Hedges *et al*. [4] demonstrated that cross-training in simulated scenarios involving 24 medical and pharmacy residents enhanced teamwork and mutual understanding. Participants were multidisciplinary pairs of residents randomly assigned to either a standard training group or a cross-training group, in which they performed tasks outside their usual scope (e.g. pharmacists intubated patients and physicians calculated pharmacokinetic doses). The results showed that cross-trained teams had significantly greater improvements in teamwork scores compared with controls. Similarly, Killeen *et al*. [14] demonstrated that virtual reality-based role swapping between surgeons and nonsurgeon team members fosters greater role awareness, empathy, and interprofessional communication. Our findings are in line with these previous observations, although applied to a different clinical setting.

The present study has certain limitations that should be acknowledged. First, the sample size is relatively small, which inevitably limits statistical power and, consequently, the robustness of the findings. This limitation affects the reliability of subgroup comparisons (nurses vs. physicians) and increases the risk of type II errors – that is, failing to reject the null hypothesis when it is false. Second, the assessment was conducted after a single endoscopy session, whereas evaluating the impact of role swapping over multiple sessions could provide a more comprehensive understanding of its effectiveness.

Third, the study was conducted in a single healthcare environment. This reduces external validity and the transferability of results to different clinical settings.

In addition, we developed a dedicated tool to assess technical skills, as there was a lack of validated, specific questionnaires. Although questionnaire validation was not the primary objective of this study, we plan to conduct both internal and external validation in future research. A larger, multicentre study is therefore warranted. Reliability will be assessed through internal consistency and by readministering the questionnaire to the same participants after an appropriate time interval has elapsed.

Finally, as a pilot study, the primary aim was exploratory, and while the results are promising, further research through larger-scale studies is needed to explore the long-term impact of role swapping on clinical outcomes and its potential scalability in other endoscopic procedures. Future studies could investigate how role swapping affects patient safety and procedural success over time, as well as whether improvements in technical and nontechnical skills translate into better real-world performance. In addition, exploring the integration of role swapping into routine clinical training programs could help determine its feasibility and effectiveness in different healthcare settings. In this context, role swapping may also have broader applications in professional development. Considering the ageing population and the anticipated rise in demand for colonoscopy, role swapping–based training could represent a valuable component in the education and upskilling of future nurse endoscopists.

### Conclusion

This pilot study provides compelling evidence that role swapping is an effective training method for enhancing technical and nontechnical skills in endoscopy. Its positive impact on role awareness, communication, teamwork, and leadership underscores its value as a helpful training tool. By fostering a more collaborative and respectful work environment, role swapping holds the potential to improve patient safety and procedural outcomes.

## Acknowledgements

We thank all the members of the Digestive Endoscopy Unit of Isola Tiberina – Gemelli Isola Hospital for actively participating in this study: Roberto Binacci, Ginevra Buccella, Luigi Cesari, Cristina Ciuffini, Elisa Colella, Carlo Covello, Giuseppe Cuccia, Marco Ferrara, Sara Gualtieri, Luciana Mancini, Maria Assunta Mancini, Omar Mariottini, Roberta Nardella, Rosella Pacioni, Marco Pantini, Luigi Giovanni Papparella, Barbara Pascucci, Martina Pontani, Giada Tacchetti, and Federica Valle.

F.B.d.A: Conceptualisation, project administration, methodology, writing (lead). S.V.d.P.: Methodology, writing. I.S.: Statistical analysis. F.M., S.F., and F.E.: Methodology. A.M.: Supervision. A.D.G.: Supervision. G.C.: Supervision, reviewing, and editing. M.B.: Supervision, reviewing, and editing.

### Conflicts of interest

G.C. is a member of the Olympus Advisory Board and the Waldner Group Advisory Board and has received support from Alfa-Sigma. For the remaining authors, there are no conflicts of interest.

## Supplementary Material


