# Supplementary material for: Role swapping between nurses and physicians in simulated endoscopy training enhances collaboration and teamwork: a prospective observational pilot study
Source: Eur J Gastroenterol Hepatol. 2025 Aug 5;38(2):154–60. doi: 10.1097/MEG.0000000000003051 (PMC12736393; doi:10.1097/MEG.0000000000003051)
Supplement: Supplementary file 1 [file ejgh-38-154-s001.docx]

**SUPPLEMENTARY MATERIALS**

| **Questions** | | **Pre** | **Post** | **p value^a^** |
| --- | --- | --- | --- | --- |
|  |  | **median (25-75th percentile)** | **median (25-75th percentile)** |  |
| Q1 | On a scale of 1-5, how confident are you in your digestive endoscopy cognitive skills? | 4 (4-4) | 5 (4-5) | 0.102 |
| Q2 | On a scale of 1-5 How confident are you in providing instructions for bowel preparation for colonoscopy? | 4 (4-5) | 5 (4-5) | 0.046 |
| Q3 | On a scale of 1-5 How confident are you in preparing the endoscopic tower? | 4 (2-5) | 5 (4-5) | **0.016** |
| Q4 | On a scale of 1-5, how confident are you in preparing the accessories and devices for the colonoscopy? | 3 (2-5) | 5 (4-5) | **0.017** |
| Q5 | On a scale of 1-5, how confident are you in preparing and diluting the drugs (adrenaline, anxiolytics/opioids, solution for submucosal injection, simethicone)? | 2 (1-3) | 4 (3-4) | **0.016** |
| Q6 | On a scale of 1-5, how confident are you in preparing the endoscopic instrument? | 4 (3-5) | 5 (4-5) | **0.026** |
| Q7 | On a scale of 1-5, how confident are you in positioning the patient for colonoscopy? | 5 (4-5) | 5 (4-5) | 0.317 |
| Q8 | On a scale of 1-5, how confident are you in setting up the audiovisual equipment (monitor and tower)? | 4 (3-5) | 5 (4-5) | 0.196 |
| Q9 | On a scale of 1-5, how confident are you in Setting up the accessories for monitoring the patient's vital signs before the procedure? | 4 (3-5) | 5 (4-5) | **0.041** |
| Q10 | On a scale of 1-5, how confident are you in anticipating the endoscopist's needs during the procedure? | 4 (3-5) | 5 (4-5) | 0.131 |
| Q11 | On a scale of 1-5, how confident are you in performing ancillary manoeuvres during colonoscopy? | 4 (3-5) | 5 (3-5) | 0.059 |
| Q12 | On a scale of 1-5, how confident are you in arranging instruments during an interventional colonoscopy? | 3 (2-5) | 5 (4-5) | **0.026** |
| Q13 | On a scale of 1-5, how confident are you in the procedure of scope disconnection from the tower at the end of the procedure? | 4 (2-5) | 5 (4-5) | 0.054 |
| Q14 | On a scale of 1-5, how confident are you in managing the patient’s discharge after the procedure? | 3 (2-4) | 5 (5-5) | **0.014** |
| Q15 | On a scale of 1-5, how confident are you in disposing of the waste after the colonoscopy? | 2 (2-3) | 5 (4-5) | **0.01** |

a: Non-parametric Wilcoxon test for paired data.

**Table 1. Confidence Levels Before and After Simulation Training in Nurse-Related Tasks.** This table presents the median confidence scores (with interquartile ranges) of endoscopists pre- and post-simulation training across various nurse-related tasks.

|  | | **Pre** | **Post** | **p value^a^** |
| --- | --- | --- | --- | --- |
|  |  | **median (25-75th percentile)** | **median (25-75th percentile)** |  |
| Q1 | On a scale of 1-5, how confident are you in your digestive endoscopy cognitive skills? | 4 (3-4) | 4 (3-4) | 0.705 |
| Q2 | How confident are you in understanding anatomy during a colonoscopy, even if altered by previous surgery? | 4 (3-4) | 4 (3-4) | 0.48 |
| Q3 | How confident are you in providing guidance on intestinal preparation for colonoscopy? | 4 (4-4) | 5 (4-5) | **0.014** |
| Q4 | How confident are you to identify the appropriateness of bowel preparation in each part of the colon, according to the Boston Bowel Preparation Scale? | 4 (3-4) | 4 (3-5) | 0.157 |
| Q5 | How confident are you in managing sedation during a colonoscopy? | 4 (4-5) | 5 (4-5) | 0.414 |
| Q6 | How confident are you in assessing appropriate indications and risks for a colonoscopy? | 4 (3-4) | 4 (3-4) | 0.655 |
| Q7 | How confident are you in identifying pathology during a colonoscopy? | 3 (2-4) | 4 (3-4) | 0.046 |
| Q8 | How confident are you in setting up therapeutic tools during a colonoscopy? | 4 (3-5) | 4 (4-5) | 0.18 |
| Q9 | How confident are you in managing complications during a colonoscopy? | 4 (2-4) | 4 (3-5) | 0.132 |
| Q10 | How confident are you in your knowledge of colonoscopy quality indicators? | 3 (2-4) | 4 (3-5) | **0.011** |
| Q11 | How confident are you in medical terminology for diseases and classifications used during colonoscopy? | 3 (2-4) | 3 (3-4) | 0.317 |
| Q12 | On a scale of 1-5, how confident are you in your endoscopy motor skills during colonoscopy? | 4 (2-5) | 4 (3-5) | **0.039** |
| Q13 | How confident are you in colonoscope insertion? | 2 (1-3) | 3 (3-4) | **0.026** |
| Q14 | How confident are you in advancing the colonoscope during the procedure? | 2 (1-2) | 3 (3-4) | **0.004** |
| Q15 | How confident are you in performing appropriate mucosal inspection during withdrawal? | 3 (1-4) | 3 (3-4) | **0.036** |
| Q16 | How confident are you in performing appropriate mucosal inspection during withdrawal? | 2 (1-4) | 3 (3-4) | **0.022** |
| Q17 | How confident are you in loop reduction during colonoscopy? | 1 (1-3) | 3 (2-4) | **0.007** |
| Q18 | How confident are you in navigating angulated turns during colonoscopy? | 1 (1-3) | 3 (2-4) | **0.007** |
| Q19 | How confident are you in performing ancillary manoeuvres during a colonoscopy? | 4 (3-5) | 5 (4-5) | **0.038** |
| Q20 | How confident are you in performing target forceps biopsy during colonoscopy? | 4 (2-5) | 4 (3-5) | 0.093 |
| Q21 | How confident are you in performing snare polypectomy on lesions smaller than 1 cm? | 3 (1-4) | 4 (3-5) | 0.088 |

a: Non-parametric Wilcoxon test for paired data.

**Table 2. Confidence Levels Before and After Simulation Training in Endoscopist-Related Tasks.** This table presents the median confidence scores (with interquartile ranges) of nurses pre- and post-simulation training across various endoscopist-related tasks.

| Question | Category | Subcategory | Question Text | Possible Answers |
| --- | --- | --- | --- | --- |
| Q1 | Situation Awareness | Gathering Information | Rate your ability to gather information during the operation. | 1 - Poor 2 - Marginal 3 - Acceptable 4 - Good 5 - Excellent |
| Q2 | Situation Awareness | Understanding Information | Rate your ability to understand the information gathered. | 1 - Poor 2 - Marginal 3 - Acceptable 4 - Good 5 - Excellent |
| Q3 | Situation Awareness | Projecting and Anticipating Future State | Rate your ability to project and anticipate the future state. | 1 - Poor 2 - Marginal 3 - Acceptable 4 - Good 5 - Excellent |
| Q4 | Decision Making | Considering Options | Rate your ability to consider alternative options. | 1 - Poor 2 - Marginal 3 - Acceptable 4 - Good 5 - Excellent |
| Q5 | Decision Making | Selecting and Communicating Option | Rate your ability to select and communicate the chosen option. | 1 - Poor 2 - Marginal 3 - Acceptable 4 - Good 5 - Excellent |
| Q6 | Decision Making | Implementing and Reviewing Decisions | Rate your ability to implement and review decisions based on changing conditions. | 1 - Poor 2 - Marginal 3 - Acceptable 4 - Good 5 - Excellent |
| Q7 | Communication and Teamwork | Exchanging Information | Rate your ability to exchange information effectively within the team. | 1 - Poor 2 - Marginal 3 - Acceptable 4 - Good 5 - Excellent |
| Q8 | Communication and Teamwork | Establishing a Shared Understanding | Rate your ability to establish a shared understanding with the team. | 1 - Poor 2 - Marginal 3 - Acceptable 4 - Good 5 - Excellent |
| Q9 | Communication and Teamwork | Coordinating Team Activities | Rate your ability to coordinate team activities during the endoscopy session. | 1 - Poor 2 - Marginal 3 - Acceptable 4 - Good 5 - Excellent |
| Q10 | Leadership | Setting and Maintaining Standards | Rate your ability to set and maintain high standards. | 1 - Poor 2 - Marginal 3 - Acceptable 4 - Good 5 - Excellent |
| Q11 | Leadership | Supporting Others | Rate your ability to support other team members effectively. | 1 - Poor 2 - Marginal 3 - Acceptable 4 - Good 5 - Excellent |
| Q12 | Leadership | Coping with Pressure | Rate the trainee's ability to cope with pressure during the endoscopy session. | 1 - Poor 2 - Marginal 3 - Acceptable 4 - Good 5 - Excellent |

**Table 3. NOTSS (The Non-Technical Skills for Surgeons) questionnaire.**
This table shows the structure of the NOTSS questionnaire, including its categories, subcategories, and corresponding questions. Responses are measured on a 5-point Likert scale ranging from 1 (Poor) to 5 (Excellent).

| Question | Category | Subcategory | Question Text | Possible Answers |
| --- | --- | --- | --- | --- |
| Q1 | Situation Awareness | Gathering Information | Do you actively monitor and collect all relevant information during the operation? | 1 - Poor 2 - Marginal 3 - Acceptable 4 - Good 5 - Excellent |
| Q2 | Situation Awareness | Recognising and Understanding Information | Do you interpret and correctly understand the information gathered during the procedure? | 1 - Poor 2 - Marginal 3 - Acceptable 4 - Good 5 - Excellent |
| Q3 | Situation Awareness | Anticipating | Do you accurately anticipate future events or complications, and foresee the surgical team's needs? | 1 - Poor 2 - Marginal 3 - Acceptable 4 - Good 5 - Excellent |
| Q4 | Communication and Teamwork | Acting Assertively | Do you demonstrate assertiveness, intervening confidently when necessary? | 1 - Poor 2 - Marginal 3 - Acceptable 4 - Good 5 - Excellent |
| Q5 | Communication and Teamwork | Exchanging Information | Do you communicate clearly and timely with the team during the operation? | 1 - Poor 2 - Marginal 3 - Acceptable 4 - Good 5 - Excellent |
| Q6 | Communication and Teamwork | Coordinating with Others | Do you effectively coordinate your actions with other members of the surgical team? | 1 - Poor 2 - Marginal 3 - Acceptable 4 - Good 5 - Excellent |
| Q7 | Task Management | Planning and Preparation | Can you plan and prepare adequately before and during the operation? | 1 - Poor 2 - Marginal 3 - Acceptable 4 - Good 5 - Excellent |
| Q8 | Task Management | Providing and Maintaining Standards | Do you maintain safety and quality standards throughout the procedure? | 1 - Poor 2 - Marginal 3 - Acceptable 4 - Good 5 - Excellent |
| Q9 | Task Management | Coping with Pressure | Do you effectively manage stress and pressure during the operation? | 1 - Poor 2 - Marginal 3 - Acceptable 4 - Good 5 - Excellent |

**Table 4. SPLINTS (Scrub Practitioners' List of Intraoperative Non-Technical Skills) questionnaire.** This table shows the structure of the SPLINTS questionnaire, including its categories, subcategories, and corresponding questions. Responses are measured on a 5-point Likert scale ranging from 1 (Poor) to 5 (Excellent).


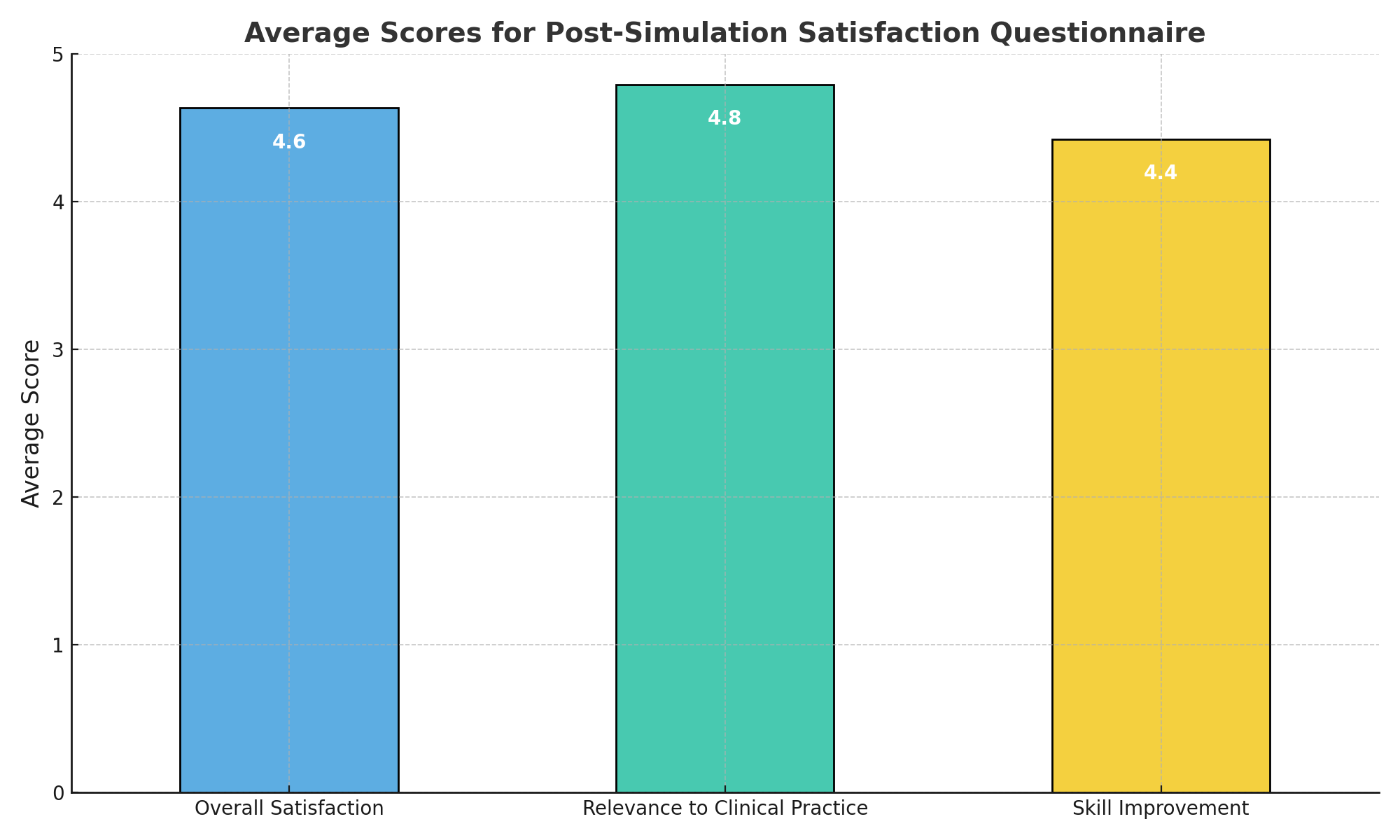


**Figure 1. Average Scores for Post-Simulation Satisfaction Questionnaire.** This figure illustrates the average scores across three dimensions: overall satisfaction, relevance to clinical practice, and skill improvement, measured on a 5-point Likert scale.
